# CPUK02 sensitizes U87 glioblastoma cell lines to TMZ treatment via autophagy flux inhibition

**DOI:** 10.22099/mbrc.2025.52011.2079

**Published:** 2026

**Authors:** Hooman Rezaie, Sanaz Dastghaib, Morvarid Siri, Pooneh Mokarram, Mina Hemmati

**Affiliations:** 1 Department of Clinical Biochemistry, School of Medicine, Zanjan University of Medical Sciences, Zanjan, Iran; 2 Endocrinology and Metabolism Research Center, Shiraz University of Medical Science, Shiraz, Iran; 3 Autophagy Research center, Shiraz University of Medical Sciences, Shiraz, Iran; 4 Autophagy Research Center, Department of Biochemistry, School of Medicine, Shiraz University of Medical Sciences, Shiraz, Iran

**Keywords:** CPUK02, TMZ, Autophagy, Glioblastoma, UPR

## Abstract

Adjuvant chemotherapy with TMZ (Temozolomide) does not improve the survival of patients suffering from GBM (Glioblastoma). Given the importance of autophagy and UPR (Unfolding Protein Response) in chemotherapy resistance, as well as the role of *Beclin-1, LC3IIβ*, and *P62* in the regulation of autophagy, we evaluated the effect of TMZ along with CPUK02 on U87 cells as a model of Glioblastoma cancer in this study. To achieve this goal, we treated the U87 cells with different doses of TMZ (50, 100, 200, 400, and 800 μM) and CPUK02 (1, 0.5, 0.25, 0.125, 0.06, 0.03, 0.01, and 0.007 μM); then, cell viability was assessed by MTT assay. The gene expression of *Beclin1, P62, LC3IIβ,* and *XBP-1s* was analyzed using quantitative real-time polymerase chain reaction. The comparison of the control group with the groups treated with the TMZ drug showed that, in 48 and 72 hours, doses of TMZ more than IC_50_ (100 μM) (*p*<0.001) significantly led to cell death. CPUK02 doses more than 0.125 (*p*<0.0001) significantly led to cell death. TMZ and CPUK02 combination therapy (100 and 0.03 μM, respectively) increased the expression of *Beclin-1, LC3IIβ*, and *P62* and activated the IRE-1 arm of UPR by increasing the expression of XBP-1s. TMZ and CPUK02 treatment inhibits the autophagic flux (*p62, LC3IIβ*). Increased *XBP-1s* expression might contribute to the enhanced TMZ sensitivity. This combination therapy is promising for TMZ-resistant cancers, but it needs further investigation.

## INTRODUCTION

Glioblastoma multiforme (GBM) is a type of glioma, a primary brain tumor originating from glial cells, and accounts for 80% of malignant CNS (Central nervous system) tumors [1]. GBM development is linked to factors like ionizing radiation, vinyl chloride, pesticides, smoking, and manufacturing-related hazards [2]. TMZ (Temozolomide) resistance complicates GBM treatment. Efforts focus on enhancing TMZ efficacy and overcoming resistance [3].

TMZ is an oral drug causing DNA damage, but survival rates for resistant patients are critically low [4, 5]. Evidence suggests that crosstalk between several pathways, including apoptosis and autophagy, and the UPR (unfolded protein response), is involved in inducing resistance to chemotherapeutic agents such as TMZ [6]. Autophagy is the recycling of cellular components in lysosomes, with three delivery methods: macro, micro, and chaperone-mediated [7]. Autophagy can support or hinder cancer cells during treatment, making it a key target for therapy [8]. Currently, the main goal of cancer treatment is to find drugs that sensitize the cells to the effect of TMZ [9].

The study found that the triple-combination therapy of TMZ, Simva (Simvastatin), and acetylshikonin significantly increased apoptosis in GBM cell lines (U87 and U251) compared to monotherapies or the dual combination of TMZ and Simva [3]. As a dynamic organelle, the endoplasmic reticulum (ER) uses the UPR—a set of intracellular signaling pathways—to regulate its protein-folding capacity in accordance with cellular requirements. [10] The UPR pathway has three arms: *ATF6* (activator transcription factor 6), *PERK* (endoplasmic reticulum RNA kinase-like kinase), and *IRE1α* (inositol-requiring enzyme 1α) [11].

*XBP-1s* (X box-binding protein 1) regulates genes for protein processing and ER expansion, aiding efficient protein production [12]. Studies indicate that Combining simva and TMZ sensitizes GBM cells to apoptosis and boosts autophagy flux via the UPR [13, 14]. Galangin, a flavonoid from honey and propolis, induces autophagy and programmed cell death, showing potential for glioblastoma combination therapy [15]. Stevia, a diterpene glycoside found in the leaves of Stevia rebaudiana, is approximately 300 times sweeter than sucrose and is widely used as a low-calorie sweetener globally [16, 17]. CPUK02 (15-Oxosteviol benzyl ester) is a diterpenoid compound with an ent-kaurane structural skeleton, which is semi-synthetically produced from stevioside. CPUK02 has been shown to induce cytotoxicity in cancer cells through the induction of apoptosis [16]. This study examined the effects of TMZ and CPUK02 co-treatment on autophagy markers and *XBP1-s* expression in U87 glioblastoma cells.

## MATERIALS AND METHODS

### Materials:

The National Cell Bank of Iran (Pasteur Institute of Iran) provided the U87 cells. CPUK02 was a generous gift from the Drug Research Institute, China Pharmaceutical University. Biosera (France) supplied the streptomycin, penicillin, FBS (Fetal bovine serum), and cell culture media. Kiyan Danesh (Shiraz, Iran) provided the RNA isolation reagent. Ampliqon (Denmark) supplied the SYBR Green PCR Master Mix. TMZ was purchased from Sigma-Aldrich Co. (Oakville, ON, Canada).

### Cell culture:

The U87 cell line, acquired from the National Cell Bank of Iran, was cultured using DMEM supplemented with 10% FBS and 1% penicillin-streptomycin. Cultures were maintained in a humidified incubator at 37°C with a 5% CO_2_ atmosphere. 

### MTT Assay:

Cell viability of TMZ and CPUK02 on U87 cells was measured using the MTT assay based on the described protocol (23). Briefly, U87 cells were seeded in 96-well plates (2 × 10^3^ cells/well) and exposed to ascending concentrations of TMZ (50, 100, 200, 400, and 800 μM) and CPUK02 (0.007, 0.01, 0.03, 0.06, 0.125, 0.25, 0.5, and 1 μM) for 48 and 72 hours. After incubation with MTT reagent (5 mg/ml, 20 μL) for 4 hours at 37°C, 100 μL DMSO was added to each well. Absorbance was measured at 570 nm using an ELISA reader (Mikura Ltd.). IC_50_ values were determined from the cell survival curves. Furthermore, the combination effect of TMZ and CPUK02 was evaluated using the MTT assay based on the optimal treatment dose and duration.

U87 cells were divided into four groups: Cells without treatment (control); Cells treated with different doses of TMZ; Cells treated with different doses CPUK02; Cells treated with the combination of TMZ and CPUK02.

### Real time PCR:

To evaluate the effect of TMZ and CPUK02 on the gene expression of Beclin-1, P62, LC3IIβ, and XBP-1s, we employed quantitative real-time polymerase chain reaction (RT-PCR). U87 cells were treated with TMZ (100 µM) and CPUK02 (0.125 and 0.03 µM) for 48 and 72 hours. Subsequently, total RNA was extracted using the Kiyan Danesh extraction reagent according to the manufacturer's protocol. cDNA was synthesized using a cDNA Synthesis Kit (Cinnagen, Iran). 

Quantities of materials used in Real-time PCR were composed of SYBR Green PCR Master Mix (5 μl), Forward primer (5 pM) (0.5 μl), Reverse primer (5 pM) (0.5 μl), distilled water (3 μl), and cDNA (1 μl). Real time PCR was performed using single-stranded cDNA. The expression level of *Beclin-1*, *P62*, *LC3IIβ*, and *XBP-1s* genes was normalized against *GAPDH*. The 2^-ΔΔct ^method was employed to compare the relative gene expression of different groups. Real-time PCR experiment was performed, using an ABI real-time PCR 7500 system. The primer sequences used in this study are shown in Table 1.

**Table 1:** The primer sequences of autophagy and UPR markers

**Genes **	**Forward primers**	**Reverse primers**
*Beclin-1 *	AGCTGCCGGTTATACTGTTCTG	ACTGCCTCCTGTGTCTTCAATCTT
*P62 *	AATCAGCTTCTGGTCCATGG	TTCTTTTCCCTCCGTGCTC
*LC3 IIβ*	AACGGGCTGTGTGAGAAAAC	AGTGAGGACTTTGGGTGTGG
*XBP-1s*	TGCTGAGTCCGCAGCAGGTG	GCTGGCAGGCTCTGGGGAAG
*GAPDH*	CGACCACTTTGTCAAGCTCA	AGGGGTCTACATGGCAA CTG

### Statistical analysis:

Statistical analysis was performed using one-way analysis of variance (ANOVA), followed by post-hoc Tukey's multiple comparison tests in GraphPad Prism 8 software. *p*-values ≤ 0.05 were considered statistically significant. All data are presented as mean ± SD. 

## RESULTS

**Figure 1:**
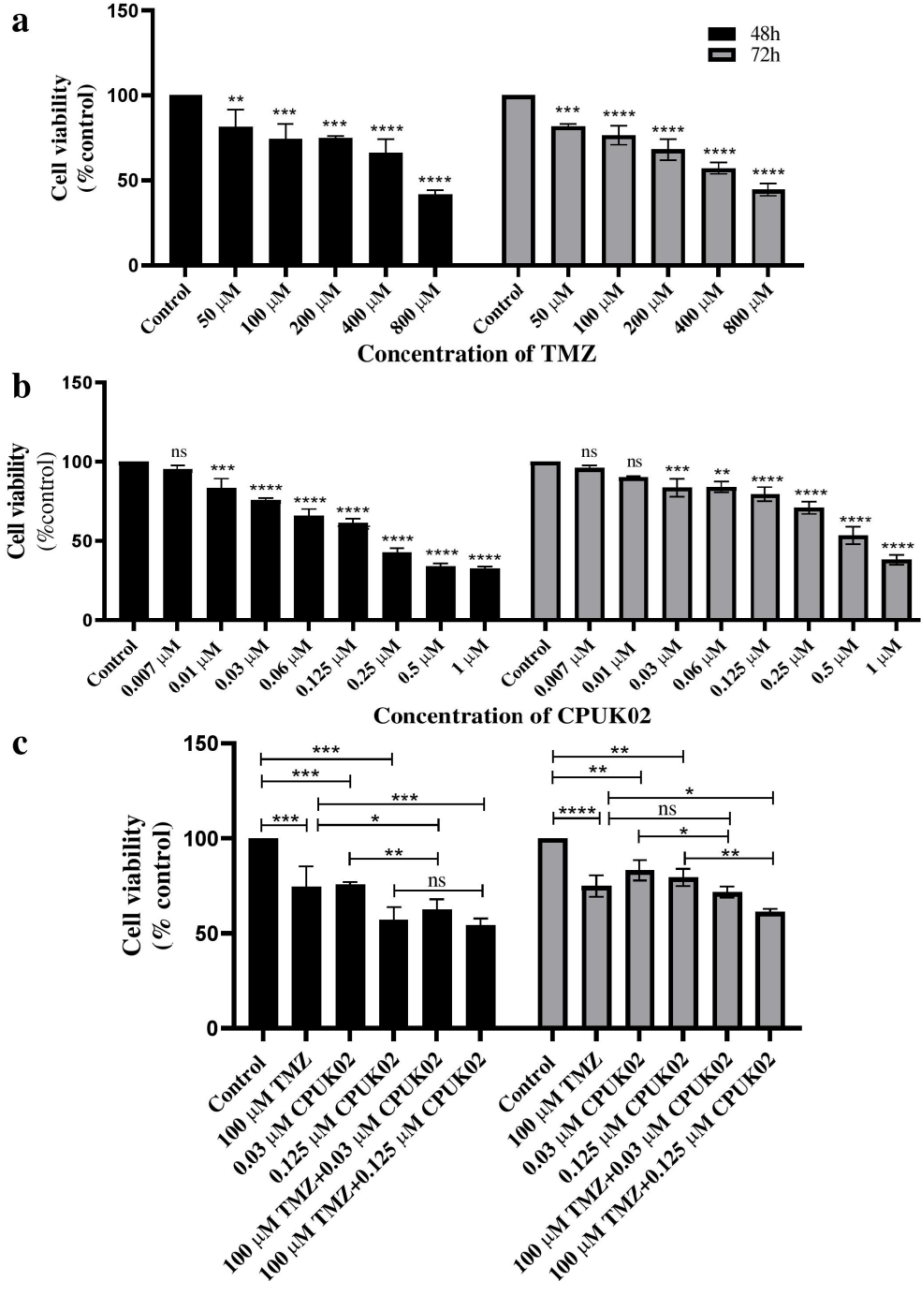
The impact of CPUK02, TMZ, and their combined application on the proliferation of U87 glioblastoma cancer cells.

Based on MTT results, doses of CPUK02 exceeding 0.01 μM exhibited a significant decrease in cell viability compared to the control group. Consequently, doses of 0.03 μM and 0.125 μM were chosen for further analysis. For TMZ, doses above 100 μM led to a significant reduction in cell viability compared to the control group. The 100 μM TMZ dose, effective for 48 and 72 hours, was selected for subsequent experiments. As shown in Figure 1c, the combination of TMZ and 0.03 μM CPUK02 induced a significant decrease in cell viability compared to 0.03 μM CPUK02 and TMZ alone. Also, CPUK02 at a dose of 0.125 μM, when combined with TMZ, considerably declined cell viability compared to TMZ alone; however, this decrease was not significant compared to 0.125 μM CPUK02 alone after 48 hours. Moreover, after 72 hours, there was a significant decrease in cell viability in 0.125 μM CPUK02 and TMZ combination group compared with 0.125 μM CPUK02 and TMZ alone (Fig. 1).

**Figure 2:**
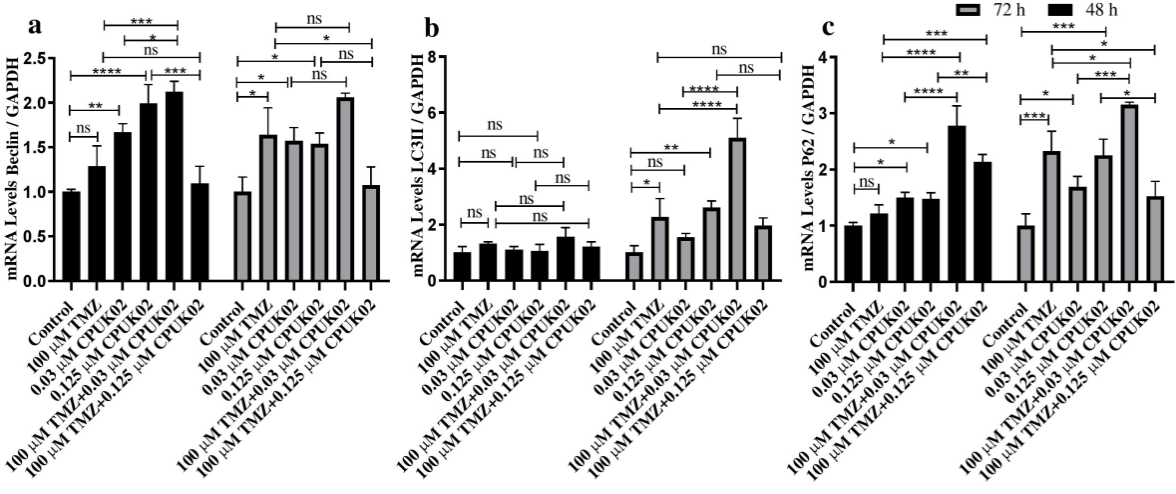
Beclin (a), LCIIβ (b), P62 (c) gene expression in 6 groups under test in 48 and 72 hours.

As shown in Figure 2a, TMZ alone did not significantly affect the increase in the *Beclin-1* expression; however, it increased the expression up to 1.6-fold at 72 hours compared to the control group. CPUK02 (0.03 μM) alone significantly increased *Beclin-1* gene expression by 1.6 times compared to the control group. Additionally, CPUK02 (0.125 μM) alone significantly increased *Beclin-1* gene expression by 1.9 times compared to the control group. The combination of TMZ with CPUK02 (0.03 μM) increased *Beclin-1* gene expression compared to 0.03 μM CPUK02 and TMZ alone at 48 hours; these changes were not significant at 72 hours. However, the combination of TMZ with CPUK02 (0.125 μM) considerably reduced the expression of this gene compared to 0.125 μM CPUK02 alone and TMZ alone.

As shown in Figure 2b, the expression of the *LC3IIβ* gene under treatment with TMZ alone did not change significantly after 48 hours but increased time-dependently by 2.2 times after 72 hours compared to the control group. CPUK02 (0.03 μM) led to a non-significant increase in *LC3IIβ* gene expression. CPUK02 (0.125 μM) increased the *LC3IIβ* gene expression, which was not significant after 48 hours but significantly increased by 2.6 times after 72 hours compared to the control group. Additionally, co-treatment with TMZ and CPUK02 did not remarkably alter the mRNA expression of *LC3IIβ* compared to TMZ and CPUK02 alone after 48 hours. However, after 72 hours, there was a considerable increase in *LC3IIβ* expression in TMZ and 0.125 μM CPUK02 combination group compared to TMZ and 0.125 μM CPUK02 alone.

Cells were treated with varying concentrations of TMZ (a), CPUK02 (b), and a combination of both (c) for 48 and 72 hours. Cell viability was subsequently evaluated using the MTT assay. The results of this assay indicate that the combined treatment of TMZ and CPUK02 induces cell death in a dose-dependent fashion. (n=3, *p<0.05; **p<0.01; ***p<0.001; ****p<0.0001).

Figure 2c demonstrates that *P62* gene expression remained relatively unchanged in the TMZ-only treated group compared to the control group at the 48-hour time but exhibited a 2.3-fold increase at 72 hours. *P62* gene expression is influenced by CPUK02. Specifically, CPUK02 at a concentration of 0.03 μM induced a slight 1.5-fold increase in *P62* gene expression. At a higher concentration (0.125 μM), this increase reached 1.5-fold after 48 hours and 2.2-fold after 72 hours compared to the control group. Co-treatment with TMZ and CPUK02 (0.03 μM) significantly upregulated *P62* expression at 48 h and 72 h compared with CPUK02 (0.03 μM) alone. TMZ and CPUK02 (0.125 μM) combination increased *P62* gene expression at 48 h but decreased it at 72 h compared to CPUK02 (0.125 μM) alone. Furthermore, as compared to TMZ alone, there was a significant upregulation of *P62* in the TMZ and CPUK02 combination group after 48 h. After 72 h, *P62* gene expression markedly elevated in TMZ and 0.03 μM CPUK02 combination group and significantly reversed in TMZ and 0.125 μM CPUK02 combination group compared to TMZ alone.

The combination of TMZ and CPUK02 (0.03 µM for 72 hours) led to increased expression of autophagy-related genes *Beclin-1*, *LC3*, and *P62*, suggesting impaired autophagic flux. *, **, ***, and **** indicate a significant difference in a, b, c gene expression with *p*<0.05, *p*<0.01, *p*<0.001, and *p*<0.0001, respectively.

**Figure 3:**
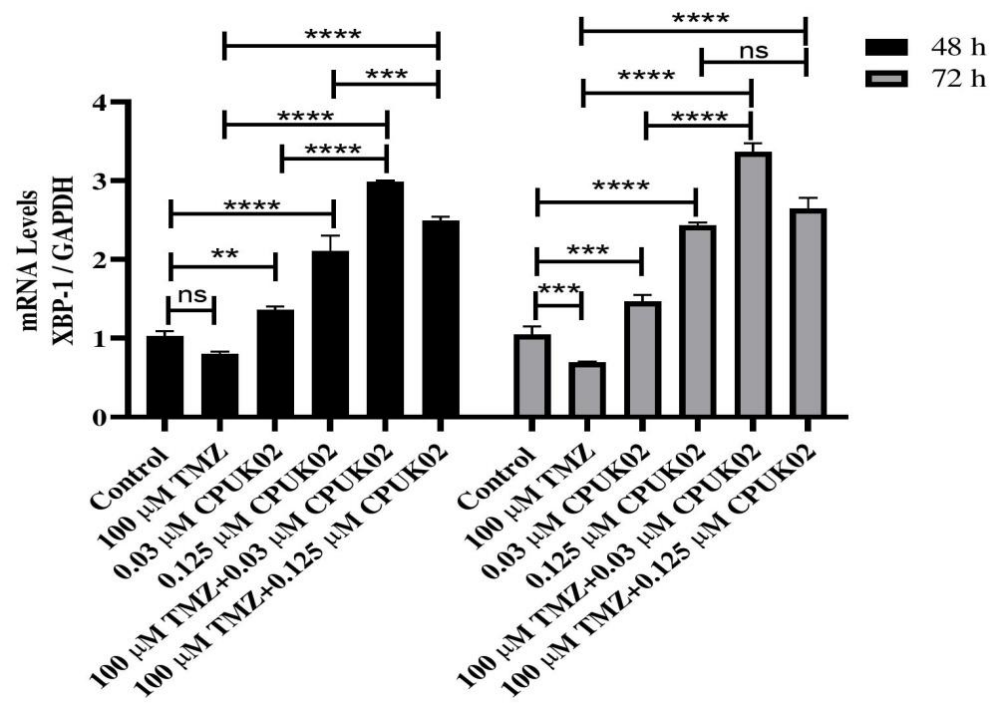
*XBP-1s* gene expression in 6 groups under test in 48 and 72 hours.

As shown in Figure 3, the expression level of the *XBP-1s* gene was determined by real-time PCR in six experimental groups. Compared to the control group, treatment with TMZ (100 μM) alone did not significantly alter *XBP-1s* gene expression at 48 hours but significantly decreased it at 72 hours. CPUK02 alone significantly increased the *XBP-1s* gene expression by 1.4 times at 0.03 μM and by 2.2 times at 0.125 μM compared to the control group. The combination of TMZ and CPUK02 (0.03 μM) significantly enhanced the *XBP-1s* expression compared to 0.03 μM CPUK02 and TMZ alone at 48 and 72 hours. Similarly, the combination of TMZ and 0.125 μM CPUK02 significantly increased the *XBP-1s* gene expression compared to 0.125 μM CPUK02 alone at 48 hours. Compared to TMZ alone, the combination of TMZ and 0.125 μM CPUK02 remarkably increased the mRNA expression of *XBP-1s* at 48 and 72 hours. 

## DISCUSSION

In this study, the combination of TMZ and CPUK02 led to an increase in autophagy markers, including *LC3-II*, *p62*, and *Beclin-1*, compared to TMZ alone in U87 human glioma cells. Additionally, the level of *XBP-1s* was also elevated. To our knowledge, this is the first report demonstrating the sensitization of glioblastoma cancer cells to TMZ chemotherapy through using CPUK02. Our findings indicated that targeting CPUK02 could be a promising strategy to augment glioblastoma cell sensitivity to TMZ via modulation of the autophagy pathway.

TMZ, CPUK02, and especially combination of TMZ and CPUK02 induced the expression of the *XBP-1*s gene in a time-dependent manner. **, *** and **** indicate a significant difference in *XBP-1s* gene expression with *p*<0.01, *p*<0.001, and *p*<0.0001, respectively.

Combining CPUK02 with TMZ in U87 glioblastoma cells increased the *Beclin-1* expression, suggesting enhanced autophagy initiation. However, elevated *P62* and *LC3-IIβ* levels after 72 hours of combined treatment with TMZ and 0.03 μM CPUK02 indicated impaired autophagy flux. MTT assay results suggest that alternative cell death mechanisms may be involved. Our study revealed alterations in autophagy-related genes, potentially inhibiting autophagic flux. Additionally, we observed increased expression of the *XBP-1s* gene, which is likely to activate the autophagy pathway. Together, these findings suggest a potential activation of apoptosis and subsequent cell death. These results demonstrate that the combined treatment effectively sensitizes the glioma cancer cells to TMZ. Based on the findings of other studies, therapeutic approaches utilizing nanomedicines and TTFields (Tumor treating fields) which may be particularly suited for combination therapy can be employed for the treatment of glioblastoma [18].

 Additionally, the use of the U251 cell line and simultaneous investigation in both U87 and U251 cell lines, while considering other pathways involved in cancer development such as *P53*, can be beneficial [19]. Additionally, increased *XBP-1s* expression, potentially inducing autophagy, might contribute to enhanced TMZ sensitivity. Furthermore, based on previous studies by Mokarram regarding the role of CPUK02 in inducing apoptosis and its positive effects in the treatment of colorectal cancer, it can be said that it could be an effective combination therapy with TMZ for sensitizing glioma cancer cells [20].

The behavior of GBM tumors in animal models varies based on their cell of origin [18]. A comprehensive treatment plan, developed by a multidisciplinary team of surgical, medical, and radiation oncologists, begins with maximal surgical resection followed by chemotherapy. For patients with methylated MGMT (O6 methylguanine DNA methyltransferase) status, the addition of TMZ (an alkylating agent) is particularly beneficial [21]. GBM cells often develop TMZ resistance after about three cycles, mainly due to elevated MGMT expression. U87 cells also show resistance [22].

 Autophagy is a cellular process that recycles damaged components to maintain balance and respond to stress [23] Like our findings on autophagy flux inhibition, this study demonstrated that GCNFs ( graphite carbon nanofibers) induced nanotoxicity in human lung cancer cells by blocking autophagy flux. This ultimately leads to apoptosis through the generation of intracellular reactive oxygen species [24] .

GBM is a highly aggressive brain tumor. Nevertheless, upon exposure to TMZ, the tumor cells enter an autophagic process for survival. Inhibiting autophagy actually induces tumor sensitivity to TMZ, thereby effectively improving treatment efficacy. The story of autophagy’s role in cancer is complicated and still being studied [25]. Chu and colleagues have further elucidated the mechanism by which thioridazine induces autophagy in GBM cells. The authors demonstrated that thioridazine upregulated the AMPK (5' AMP-activated protein kinase) activity and, similar to our findings, increased *p62-*mediated autophagy, which can inhibit autophagy flux and apoptosis via the Wnt /β-catenin signaling pathway [26].

Seung Woo et al. showed that combining TMZ (50 μM) and CQ (chloroquine) inhibits glioma cell growth via autophagy and has effects influenced by *P53* status, using the U87 cell line for comparison [27]. In contrast to our findings, another study proposed that inhibiting *Beclin-1* could suppress autophagy and potentially overcome drug resistance. Interestingly, this study reported a significant decrease in both autophagy markers, *LC3-II*, and *Beclin-1*, in HCT116 colon cancer cells following 5-FU (5-Fluorouracil) treatment [20].

Johannessen et al.'s study, similar to ours, reported on thioridazine (an antipsychotic drug) disrupting *LC3I/II* and *P62* accumulation (markers of autophagy inhibition), compromising new autophagy and leading to enhanced TMZ sensitivity [28] In another study, Wang et al. (2020) showed that autophagy inhibition in breast cancer cells led to *p62* accumulation and subsequent ubiquitin-protein aggregate formation which can denote the initiation of apoptosis [29]. 

 In some instances, the combination of bevacizumab with other chemotherapeutic agents like carboplatin, irinotecan, etoposide, erlotinib, and cetuximab has not shown a significant improvement in survival compared to bevacizumab monotherapy.[30-32]. Lovastatin enhances the anti-cancer efficacy of temozolomide by suppressing autophagy flux in temozolomide-resistant glioblastoma cells.[33] Consistent with our findings, in another study by Song et al. (2019), by inhibiting autophagy flux, temozolomide-perillyl alcohol conjugate (NEO212) induced G2/M phase cell cycle arrest, mitochondrial fragmentation, DNA damage, and apoptosis in ovarian cancer cells. The agent demonstrates cytotoxic activity against malignant cells.[34].

In-vitro studies show that a semi-synthetic stevioside called CPUK02 inhibits the proliferation of human cancer cell lines, suggesting potent anticancer activity. At lower concentrations, it exhibited a preferential cytotoxic effect for cancer cells vs normal liver cells. Its antitumor activity has been confirmed in-vivo in human xenograft tumor models, comparable to the chemotherapeutic agent 5-FU but with a superior toxicity profile. Mechanistic studies are under way to better understand its specific anticancer mode of action [16].

CPUK02 (1,6,8,10,16,32 and 64 μM in two cell lines) treatment inhibited the UPR and modulated autophagy by decreasing *Beclin-1* and increasing *P62* and *LC3βII* mRNA levels in colorectal cancer cells. These findings suggest that CPUK02 exerts its cytotoxic effects by disrupting these essential cellular processes [20]. During metastasis, the *IRE1α-XBP1* pathway interacts with *HIF1α* (Hypoxia-inducible factor 1-alpha) to promote tumor growth and survival in hypoxia, making it a potential target for treating metastatic cancer [35]. The combination of Simva and TMZ activates the UPR pathway, inhibits autophagy, and increases the expression of UPR-related genes like *XBP-1s*. High UPR activation may lead to apoptosis and cell death, suggesting a similar mechanism in this study [14]. 

CPUK02 demonstrated strong anticancer effects, effectively inhibiting proliferation and inducing apoptosis in various human cancer cell lines. In a mouse xenograft model, research by sun and colleagues found CPUK02 to outperform 5-FU in antitumor activity [36] . Despite the focus of the study on the potential effects of CPUK02 on the sensitivity of U87 glioma cells via targeting autophagy, several limitations should be acknowledged. The focus of the current study is solely on the U87 cell line, a mutated glioma cell line frequently used in research. In future studies, it will be important to use additional glioma cell lines with different characteristics to more accurately evaluate the specific effects of CPUK02. To strengthen the conclusion—that one of the pathways affected by this compound is autophagy—it is essential to examine other glioblastoma cell lines in future studies. While qRT-PCR was used to measure mRNA levels of autophagy markers in this study, future research should include Western blot analysis to validate these findings. Furthermore, quantifying protein levels in each signaling pathway would provide a more complete picture and strengthen the conclusions.

Combined TMZ and CPUK02 significantly enhanced the sensitivity of U87 glioma cells to chemotherapy compared to TMZ alone. This study provides the first evidence that CPUK02 can potentiate the anti-tumor effects of TMZ. Mechanistically, while CPUK02 increased autophagy initiation, impaired autophagy flux was observed. Additionally, upregulated *XBP-1s* suggests a potential role for ER stress in the observed effects. Targeting CPUK02 emerges as a potential therapeutic avenue for glioblastoma, with the possibility of synergistic effects when combined with the current standard treatment, TMZ

### Acknowledgements

The authors would like to thank Shiraz University of Medical Sciences, Shiraz, Iran and also Center for Development of Clinical Research of Nemazee Hospital and Dr. Nasrin Shokrpour for editorial assistance.

### Conflict of Interest:

The authors declare that they have no conflict of interest.

### Ethics approval and consent to participate:

This study was approved by the vice-chancellor of research and technology, and ethics committee of Zanjan University of Medical Sciences [ethical code: IR.ZUMS.BLC.1402.015]. Ethical considerations were observed in all steps of the research.

### Authors’ Contribution:

HR, MS did all experiments, figures, and table preparation, and prepared the first draft of the manuscript. SD set up all experiments and prepared the second draft of the manuscript. PM co-correspond to the project, made the initial plan of the project, supervised the direction of the project, and did a final proof of the manuscript. MH co-correspond to the project, made the final plan for the project, supervised the direction of the project, and did a final proof of the manuscript. All authors have read and agreed to the published version of the manuscript.
